# Genetic testing and human leukocyte antigen in patients with hypertrophic cardiomyopathy and connective tissue diseases

**DOI:** 10.3389/fgene.2024.1432670

**Published:** 2024-08-06

**Authors:** Daigo Hiraya, Nobuyuki Murakoshi, Miyako Igarashi, DongZhu Xu, Tomoko Ishizu

**Affiliations:** Department of Cardiology, Institute of Medicine, University of Tsukuba, Tsukuba, Japan

**Keywords:** hypertrophic cardiomyopathy, genetic mutations, human leukocyte antigen, rheumatoid arthritis, connective tissue diseases

## Abstract

Hypertrophic cardiomyopathy (HCM) is caused by myocardial hypertrophy, often due to mutations in cardiac sarcomere protein genes such as beta-myosin heavy chain (MYH7) and myosin-binding protein C (MYBPC3). However, a significant proportion of HCM cases lack identified genetic mutations, and genotype-phenotype correlations remain unclear. Concurrently, potential associations between HCM and human leukocyte antigen (HLA) types, as well as connective tissue diseases, have been proposed. In this single-center study, we aimed to investigate the genetic and HLA profiles of patients with obstructive hypertrophic cardiomyopathy (HOCM) and connective tissue diseases, particularly focusing on the prevalence of genetic variants and HLA types. We conducted a detailed analysis of five patients with HOCM and connective tissue diseases and sarcoidosis, identifying rare variants in causative genes for HCM in two cases and observing specific HLA types that were relatively common. Notably, 15% of all HOCM cases presented with connective tissue diseases, mainly rheumatoid arthritis. These findings underscore the complexity of HCM etiology and suggest potential implications for both diagnostic strategies and therapeutic approaches in patients with concomitant inflammatory conditions.

## 1 Introduction

Genetic testing is central to understanding the pathogenesis of hypertrophic cardiomyopathy (HCM), with more than 2000 mutations identified in over 11 sarcomere genes (Authors/Task Force members et al., 2014; Kitaoka et al., 2021). Mutations in the genes encoding the beta-myosin heavy chain (MYH7) and myosin-binding protein C (MYBPC3) account for the majority mutations. However, the detection rate of known pathogenic mutations in HCM patients without a family history or in older patients is only 30%–40%, highlighting significant gaps in our understanding (Bonaventura et al., 2021). Furthermore, genotype-phenotype correlations remain inconsistent, underscoring the need for further investigation (Marian and Braunwald, 2017; Maron, 2018). There is emerging evidence suggesting an association between obstructive hypertrophic cardiomyopathy (HOCM) and human leukocyte antigen (HLA), indicating potential links between immune responses and HCM. HLA genes encode major histocompatibility complex (MHC) proteins that play a crucial role in immune responses and have been implicated in various diseases, including autoimmune and chronic inflammatory conditions (Choi et al., 2021). There are many types of HLA antigens, of which HLA-A, HLA-B, and HLA-DRB1 are highly polymorphic (Tokunaga, 2014). The involvement of HLA-DR4 and HLA-DR1 in connective tissue diseases, particularly rheumatoid arthritis (RA), has been previously reported (Roudier, 2000). Several studies have demonstrated the involvement of HLA-DR4 in patients with HOCM combined with RA and mixed connective tissue diseases (Nakamura et al., 2008; Dawood et al., 2018). Despite these insights, there is limited research exploring the involvement of HLA and concomitant inflammatory conditions in HCM. Previous studies have hinted at the presence of “inflammatory hypertrophic cardiomyopathy”, suggesting a novel avenue for investigation. However, the precise role of HLA and its implications in HCM remain largely unexplored. Therefore, this study aims to address this gap in the literature by investigating the role of HLA and performing genetic testing in patients with HCM and connective tissue diseases.

## 2 Materials and methods

On 14 February 2024, we investigated the prevalence of connective tissue diseases in 61 patients with HOCM who had outpatient appointments at the University of Tsukuba Hospital, Tsukuba, Japan. HOCM was defined as follows: i) maximum left ventricular wall thickness ≥15 mm; ii) cardiac magnetic resonance imaging (MRI) or endomyocardial biopsy ruling out secondary cardiomyopathy; and iii) left ventricular pressure gradient ≥30 mmHg at rest or during physiological provocation (Kitaoka et al., 2021). A rheumatologist made a definitive diagnosis of connective tissue diseases.

### 2.1 HLA analysis

Venous blood (5 mL) was collected in tubes containing ethylenediaminetetraacetic acid (EDTA) and used either fresh or frozen. The samples were sent to an external institution (LSI Medience Co., Tokyo, Japan) for human leukocyte antigen (HLA) genotyping. The genotyping was conducted using a reverse sequence-specific oligonucleotide probe protocol with a Luminex 100 × MAP flow cytometry dual-laser system, following the polymerase chain reaction (PCR)-Luminex method (Itoh et al., 2005; Azuma, 2021). Sets of polystyrene color-coded microbeads (Multi-Analyte Microsphere Carboxylated; Luminex, Austin, TX, USA), approximately 5.5 μm in diameter, were labeled with oligonucleotide annealing probes as part of the xMAP technology. Each set of beads was color coded by the manufacturer using a specific ratio of two different fluorescent dyes (red and infrared) embedded in the beads. Up to 100 different fluorescently labeled microbeads were coded and identified using the Luminex 100 flow cytometer by adjusting the concentration of each fluorochrome. One of the dual lasers on the Luminex 100 excited the internal dyes within the beads, identifying the exact code number of the fluorescent microbeads by determining the preset ratio of the internalized dyes. Target DNA was PCR-amplified using 5′-biotin-labeled primers highly specific to certain sequences of HLA genes. After denaturation at 95°C, amplified DNA hybridized to complementary DNA probes coupled to microbeads. The hybridized PCR product on the oligobeads was labeled with streptavidin-phycoerythrin. The Luminex apparatus was used to identify the fluorescence intensity of phycoerythrin on each coded oligobead hybridized with the biotin-labeled PCR product. Genosearch typing software assisted in determining the HLA genotype (alleles) of the sample DNA. HLA-DRB1*04 was serologically identified as HLA-DR4, and HLA-DRB1*01 was identified as HLA-DR1.

### 2.2 Genetic testing

The genetic analysis was reviewed and approved by the University of Tsukuba Clinical Research Ethics Review Committee (approval number: R02-300). After obtaining written informed consent from the patients, next-generation sequencing was conducted using the Ion Proton System (Thermo Scientific, Waltham, Massachusetts, USA) with the Ion AmpliSeq™ Cardiovascular Research Panel and the Ion AmpliSeq™ Library Kit 2.0. Primary processing of reads utilized Ion Proton Software (Thermo Fisher Scientific). Subsequent steps involved alignment with the human reference genome (GRCh38–hg19), base calling, trimming, and filtering of poor signal reads using the Ion Torrent Software Suite (ISS) version 5.4.0. The VCF file was uploaded and annotated using the wANNOVAR software. Variants classified as pathogenic or likely pathogenic for HCM according to ClinVar were considered causative genes and were confirmed by direct sequencing.

### 2.3 Endomyocardial biopsy

An endomyocardial biopsy was performed to exclude secondary cardiomyopathy. Myocardial specimens were collected from the right ventricular septum via the internal jugular venous approach using a 7-Fr bioptome (Cordis; Johnson and Johnson Co., New Brunswick, NJ, USA). At least three specimens were procured, with one undergoing electron microscopic evaluation and the others subjected to light microscopic examination. Biopsy samples for light microscopy analysis were transferred from the bioptome to a fixative (10% neutral buffered formalin), embedded in paraffin, and sectioned into 3-μm-thick sections. These sections were sequentially stained with hematoxylin and eosin, Masson’s trichrome, and Congo red (Yamamoto et al., 2023).

## 3 Results

Among the 61 patients with HOCM, 9 (15%) had connective tissue disease (5 with RA, 1 with systemic lupus erythematosus, 1 with scleroderma, 1 with Takayasu arteritis, and 1 with sarcoidosis). HLA analysis and genetic testing were conducted in five of these patients (Table 1). Only two of the five patients with a family history had rare variants in HCM causative genes (case 1 and 2). In HLA analysis, case 2 had HLA-DR4, case 4 and 5 had HLA-DR1, case 1, 3 and 5 had HLA-A26, case 2, 4 and 5 had HLA-B7, and case 2, 3 and 5 had HLA-DR9, respectively.

**TABLE 1:** List of patients with obstructive hypertrophic cardiomyopathy and chronic inflammatory diseases.

Case no.	Age, sex	Family history of HCM	Maximum LV wall thickness (mm)	LV-PG (mmHg)	Types of chronic inflammatory diseases (disease duration)	Medication	CRP (mg/dL)	Pathological findings	HLA types	Genetic testing
1	44, F	Mother Grandmother	19	126	RA (10 years)	MTX ETN	1.19	NA	A*11, A*26 B*15, B*67 DRB1*14, DRB1*15	MYH7(NM_000257.4): c.1870T>A: p.Tyr624Asn
2	59, F	Mother	19	122	RA (3 years)	MTX	0.26	Hypertrophy and disarray of cardiomyocytes, interstitial fibrosis	A*24, -B*07, B*40 DRB1*04, DRB1*09	MYL2(NM_000432.4): c.173A>G: p.Arg58Gln
3	61, F	None	22	62	RA (29 years)	PSL MTX TAC	0.09	NA	A*26, A*31 B*40, B*48 DRB1*09, DRB1*14	Under analysis
4	69, F	None	15	72	RA (9 years) PM (1 year)	PSL	2.10	Nuclei size differences in cardiomyocytes, interstitial fibrosis	A*02, A*11 B*07, B*67 DRB1*01, DRB1*14	No pathogenic variants
5	68, F	None	17	69	Sarcoidosis	PSL	0.85	Interstitial fibrosis	A*24, A*26 B*07, B*15 DRB1*01, DRB1*09	No pathogenic variants

CRP = C-reactive protein; ETN = etanercept; F = female; HCM = hypertrophic cardiomyopathy; HLA = human leukocyte antigen; LV = left ventricular; MTX = methotrexate; MYH7 = ; NA = not available; PG = pressure gradient; PM = polymyositis; PSL = prednisolone; RA = rheumatoid arthritis; TAC = tacrolimus.

Two cases (case 1 and 2) are described in detail.

### 3.1 Case 1

**FIGURE 1:**
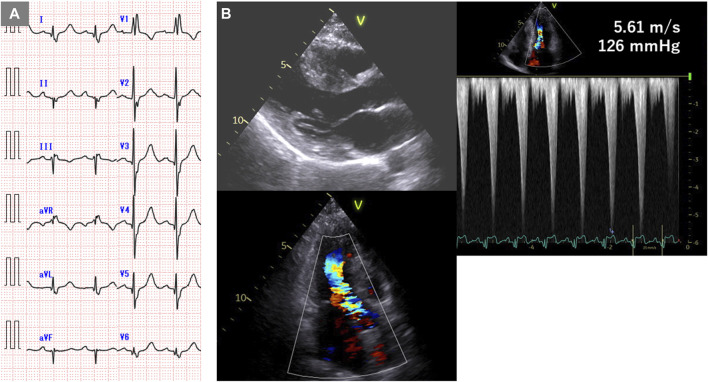
Electrocardiogram and echocardiogram of case 1. **(A)** Electrocardiogram showing complete right bundle branch block and left anterior hemiblock. **(B)** Echocardiogram showing mid-septal wall thickness of 19 mm and resting pressure gradient of 126 mmHg.

**FIGURE 2:**
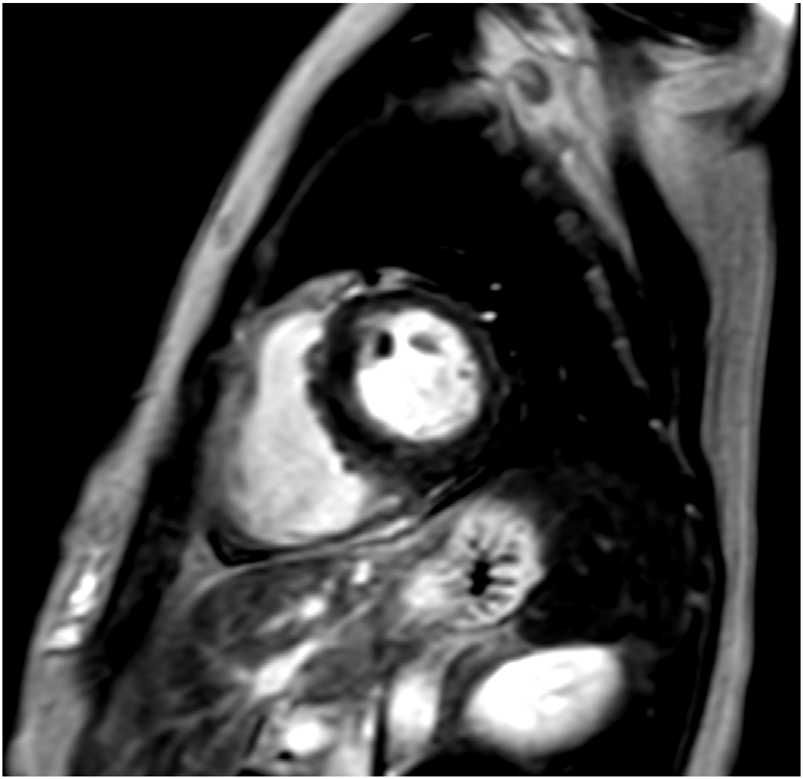
Cardiac magnetic resonance imaging (MRI) of case 1. Cardiac MRI showing no late gadolinium enhancement (LGE).

The patient, a 44-year-old woman, had a familial history of sudden death from HCM (Ohsuzu et al., 1997). Electrocardiography revealed a complete right bundle branch block and left anterior hemiblock (Figure 1A). The mid-septum wall thickness was 19 mm, with a resting pressure gradient of 126 mmHg from the mid-ventricle to the outflow tract (Figure 1B). Diagnosed with RA at 34 years, the patient was treated with methotrexate and etanercept. Serum CRP levels were elevated at 1.19 mg/dL. Cardiac magnetic resonance imaging (MRI) showed no late gadolinium enhancement (LGE) (Figure 2), and no endomyocardial biopsy was performed. HLA-DRB1*14 and HLA-DRB1*15 were identified. Previous genetic testing revealed a missense variant (c.1870T>A, p. Y624N) in the MYH7 gene (NM_000257.4). This variant has been reported in an HCM cohort, and is not present in population databases. Therefore, this is predicted to be a pathogenic variant causative for HCM, but has been reported as VUS in ClinVar.

### 3.2 Case 2

**FIGURE 3:**
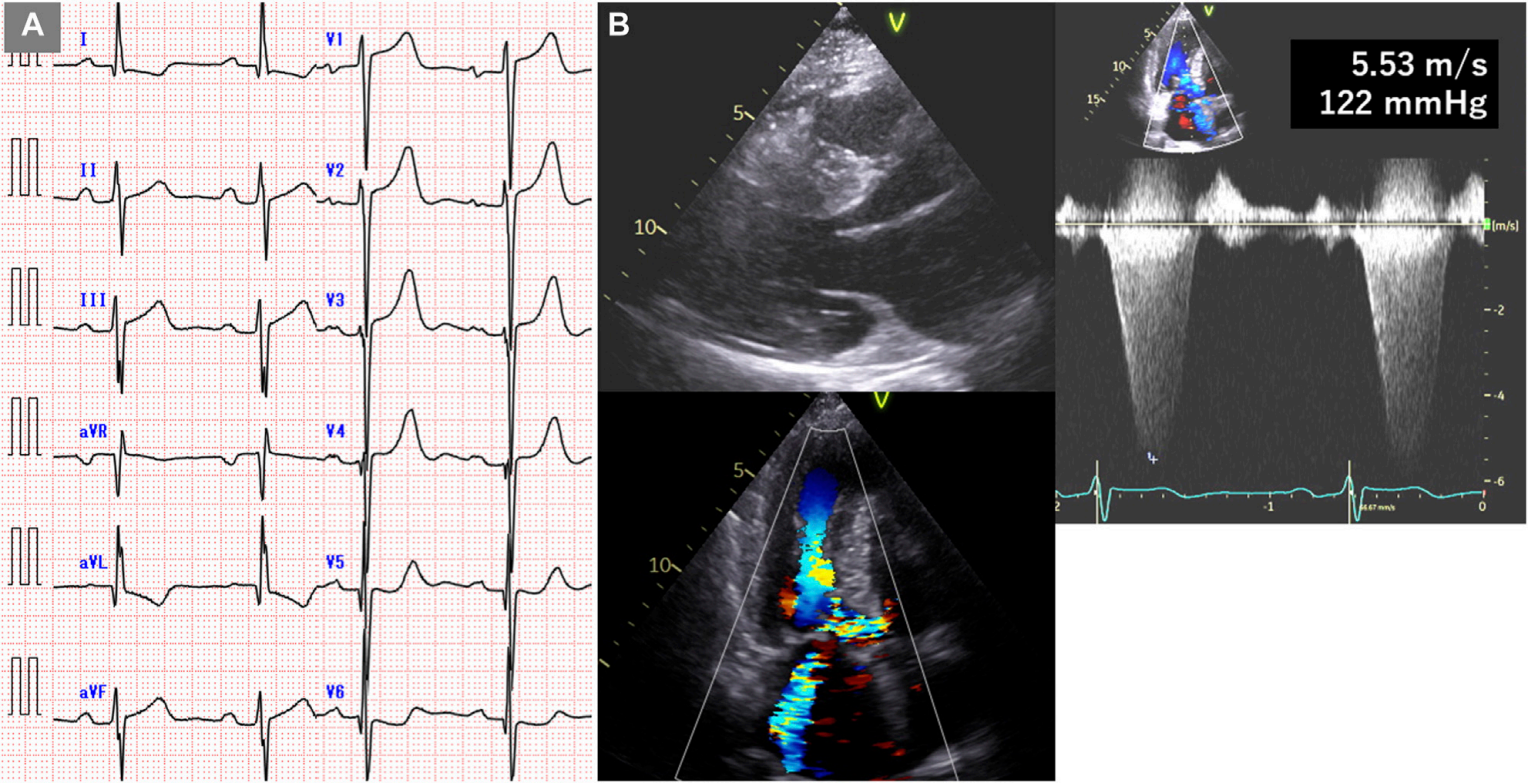
Electrocardiogram and echocardiogram of case 2. **(A)** Electrocardiogram showing grade I atrioventricular block, left axis deviation, and ST-T abnormality. **(B)** Echocardiogram showing mid-septal wall thickness of 19 mm and resting pressure gradient of 122 mmHg.

**FIGURE 4:**
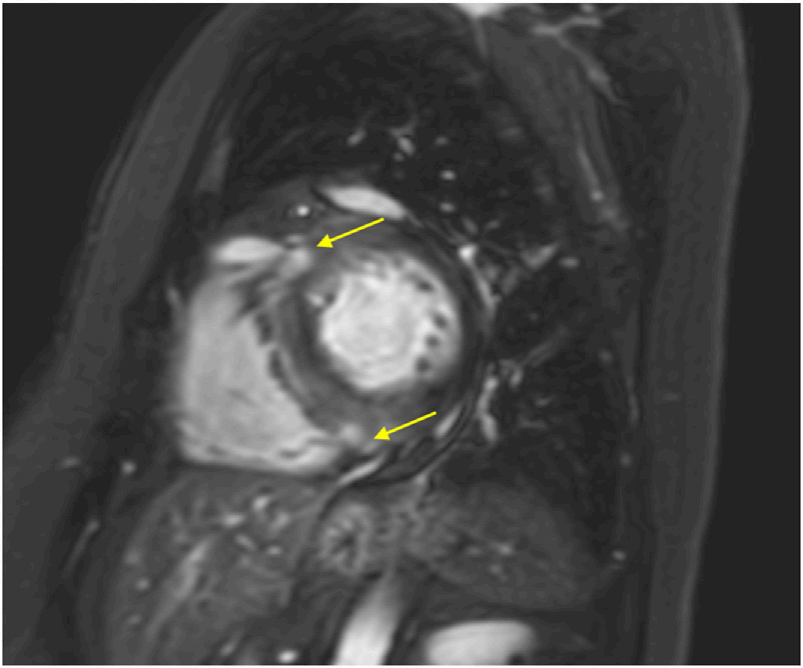
Cardiac MRI of case 2. Cardiac magnetic MRI showing LGE in the septum at the right ventricular junction (yellow arrows).

**FIGURE 5:**
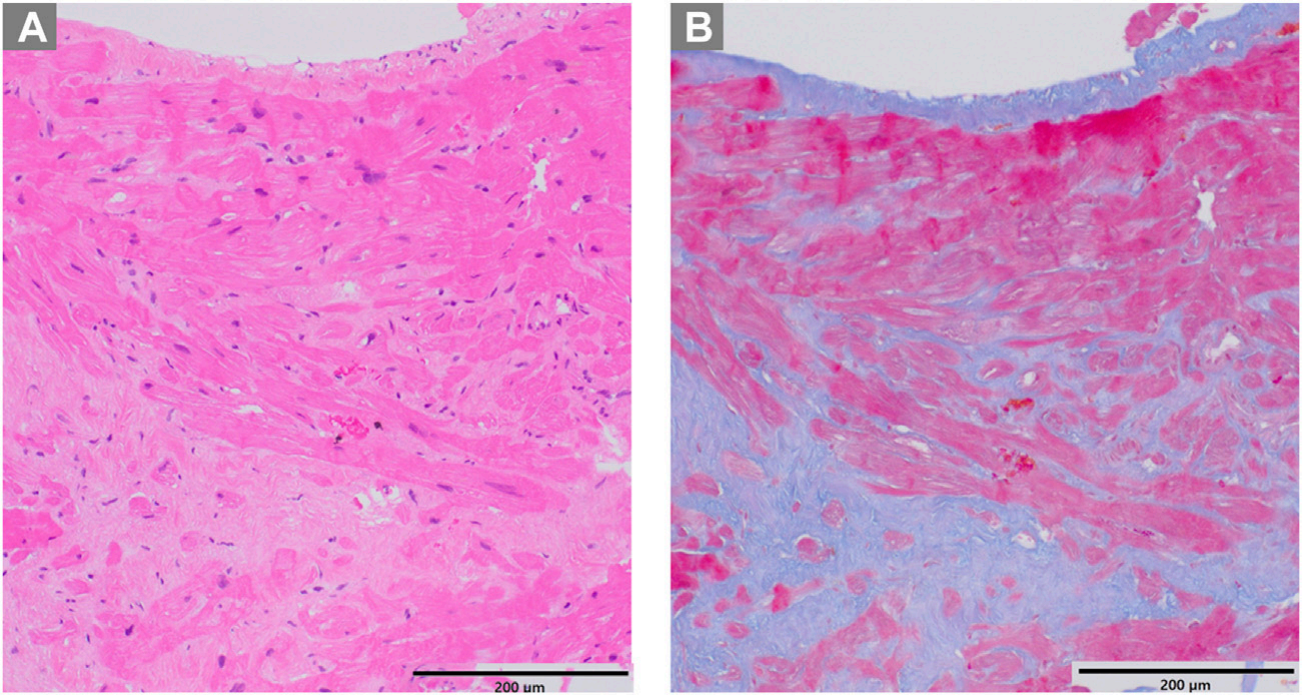
Pathological examination of case 2. **(A)** Hematoxylin-eosin staining revealing hypertrophy and disarray of myocardial cells **(B)** Masson trichrome staining showing interstitial fibrosis.

The patient, a 59-year-old woman with a family history of cardiac hypertrophy, exhibited a grade I atrioventricular block, left axis deviation, and ST-T abnormality on electrocardiography (Figure 3A). The mid-septum wall thickness measured 19 mm, with a resting pressure gradient of 122 mmHg observed in the left ventricular outflow tract (Figure 3B). Diagnosed with RA at 56 years old, the patient underwent methotrexate treatment, while her serum CRP level was slightly elevated at 0.26 mg/dL. Cardiac MRI revealed LGE in the septum of the right ventricular junction (Figure 4). Pathological examination revealed hypertrophy, myocardial cell disarray, and interstitial fibrosis (Figure 5), with no observed inflammatory cell infiltration. HLA-DRB1 types were determined as HLA-DRB1*04 and HLA-DRB1*09, while genetic testing revealed a missense variant (c.173G>A, p. R58Q) of the myosin light chain 2 (MYL2) gene (NM_000432.4), which has been classified as pathogenic for HCM.

## 4 Discussion

A previous study reported the prevalence of RA in Japan to be 0.6%–1.0% (Yamanaka et al., 2014). In this study, out of 61 patients with HOCM, 9 (15%) had connective tissue disease, and five (8%) of them had RA. These results suggest a close relationship between HOCM and chronic inflammation. HLA is directly involved in antigen presentation, self- and non-self-identification. The HLA-class I molecules, HLA-A, HLA-B, and HLA-C, are expressed on the cell membranes of all nucleated cells and platelets. They identify non-self-endogenous peptides and stimulate CD8^+^ killer T cells. HLA class II molecules, HLA-DR, HLA-DQ, and HLA-DP, are expressed on the membranes of antigen-presenting cells such as monocytes, macrophages, dendritic cells, and B cells, and identify exogenous peptides that activate CD4^+^ helper T cells (Ogawa, 2016a).

Several studies have reported the presence of HLA-DR4 in patients with HOCM and connective tissue diseases. The involvement of HLA-DR4 and HLA-DR1 in RA has been previously reported. In this study, one of the five patients had HLA-DR4 (case 2), and two had HLA-DR1 (cases four and 5). HLA-DRB1, a major risk allele for RA, stimulates CD4^+^ helper T cells, which enhances the production of interleukin-17 and interferon-γ, leading to chronic inflammation (Eid et al., 2009). No significant inflammatory cell infiltration was observed in the myocardial biopsy samples analyzed in this study. This is probably because myocardial hypertrophy is induced by chemokines and cytokines due to systemic inflammation rather than by inflammatory cell infiltration into the myocardium itself. In RA, transforming growth factor β (TGF-β) is secreted from fibroblast-like synoviocyte during a series of immune and inflammatory processes (Mclnnes and Schett, 2011). Because TGF-β stimulates cardiac microvascular endothelial cells and is involved in their conversion from endothelial to mesenchymal cells and migration into the myocardium, TGF-β signaling is a crucial mechanism for increases fibrosis in HCM. Treatment of HCM mice with TGF-β-neutralizing antibodies markedly reduces the expression of periostin, which promotes differentiation into fibroblasts and improves cardiac hypertrophy (Teekakirikul et al., 2010). Moreover, JAK/STAT and MAPK pathways, which are activated in RAs and are important for disease progression, also play a major role in the progression of cardiac hypertrophy. The close involvement of common pathways in disease progression may be one of the mechanisms by which HCM occurs in patients with RA.

In this study, we found that three of the five patients had HLA-A26, HLA-B7, HLA-DR9, and/or HLA-DR14, suggesting that HOCM may be associated with some HLA types, although the number of cases was small. Further investigation is required to confirm these findings.

Patients in this study received treatment for connective tissue diseases using prednisolone or methotrexate. While steroids are associated with the development of cardiovascular disease, anti-rheumatic drugs like methotrexate, tumor necrosis factor (TNF) inhibitors, and IL-6 inhibitors have been reported to mitigate this risk (Crowson et al., 2013; Giachi et al., 2022). Controlling inflammation may aid in suppressing myocardial hypertrophy and fibrosis in patients with HCM complicated by connective tissue diseases. In addition, T cells and TGF-β could potentially emerge as future therapeutic targets. However, anti-inflammatory treatment mechanisms are intricate, necessitating consideration of cytokine and chemokine interaction networks, rather than individual pro-inflammatory factors.

This study had several limitations. Firstly, it was a small, single-center study, and detailed genetic testing is ongoing; therefore, it remains unclear whether known genetic mutations are implicated in patients with HCM and connective tissue diseases. Secondly, due to racial and regional variations in HLA phenotypes, the results of this study cannot be generalized worldwide. Thirdly, the highly polymorphic nature of the HLA gene necessitates clarification of the odds ratio to establish its association with HCM (Ogawa, 2016b). Furthermore, a detailed nucleotide sequence investigation of HLA was not conducted, precluding determination of whether specific HLA alleles or antigens contribute to HCM or other connective tissue diseases. Lastly, the deposition of amyloid A protein in the myocardium due to chronic inflammation was not investigated. While our study provides valuable insights into the association between HCM and concomitant inflammatory diseases and proposes the existence of a clinical condition called “inflammatory HCM”, it is important to acknowledge certain limitations that may affect the interpretation and generalizability of our findings. Further discussion regarding the potential impact of these limitations on the reliability and applicability of our results could provide with a clearer understanding of the study scope and implications.

To address the limitations of our study and further elucidate the relationship between HCM and HLA, future research endeavors could focus on conducting larger, multicenter studies involving diverse racial and regional populations. In addition, detailed investigations into the deposition of amyloid A protein in the myocardium and comprehensive nucleotide sequence analyses of HLA genes could provide valuable insights into the underlying mechanisms linking chronic inflammation to HCM. If the association between HCM and connective tissue diseases is confirmed, it is expected to advance not only diagnostic and preventive medicine but also the development of novel immune system-targeted treatments.

## Data Availability

The datasets presented in this article are not readily available because they contain personal genetic information and have not been approved in our ethical review for inclusion in publicly available databases. Requests to access the datasets should be directed to the correspondence (d.hiraya@md.tsukuba.ac.jp).

## References

[B1] Authors/Task Force members Elliott P. M. Anastasakis A. Borger M. A. Borggrefe M. Cecchi F. Charron P. (2014). 2014 ESC guidelines on diagnosis and management of hypertrophic cardiomyopathy: the task Force for the diagnosis and management of hypertrophic cardiomyopathy of the European society of cardiology (ESC). Eur. Heart J. 35 (39), 2733–2779. 10.1093/eurheartj/ehu284 25173338

[B2] Azuma F. (2021). Overview of HLA DNA typing technology. Major Histocompat. Complex 28 (2), 83–93. 10.12667/mhc.28.83

[B3] Bonaventura J. Polakova E. Vejtasova V. Veselka J. (2021). Genetic testing in patients with hypertrophic cardiomyopathy. Int. J. Mol. Sci. 22 (19), 10401. 10.3390/ijms221910401 34638741 PMC8509044

[B4] Choi W. Luo Y. Raychaudhuri S. Han B. (2021). HATK: HLA analysis toolkit. Bioinformatics 37 (3), 416–418. 10.1093/bioinformatics/btaa684 32735319 PMC8058762

[B5] Crowson C. S. Liao K. P. Davis J. M. Solomon D. H. Matteson E. L. Knutson K. L. (2013). Rheumatoid arthritis and cardiovascular disease. Am. Heart J. 166 (4), 622–628. 10.1016/j.ahj.2013.07.010 24093840 PMC3890244

[B6] Dawood M. Lateef N. Tauseef A. Patel J. (2018). Association of hypertrophic obstructive cardiomyopathy with rheumatoid arthritis. Cureus 10 (1), e2028. 10.7759/cureus.2028 29531881 PMC5837261

[B7] Eid R. E. Rao D. A. Zhou J. Lo S. F. Ranjbaran H. Gallo A. (2009). Interleukin-17 and interferon-gamma are produced concomitantly by human coronary artery-infiltrating T cells and act synergistically on vascular smooth muscle cells. Circulation 119 (10), 1424–1432. 10.1161/CIRCULATIONAHA.108.827618 19255340 PMC2898514

[B8] Giachi A. Cugno M. Gualtierotti R. (2022). Disease-modifying anti-rheumatic drugs improve the cardiovascular profile in patients with rheumatoid arthritis. Front. Cardiovasc Med. 9, 1012661. 10.3389/fcvm.2022.1012661 36352850 PMC9637771

[B9] Itoh Y. Mizuki N. Shimada T. Azuma F. Itakura M. Kashiwase K. (2005). High-throughput DNA typing of HLA-A, -B, -C, and -DRB1 loci by a PCR-SSOP-Luminex method in the Japanese population. Immunogenetics 57 (10), 717–729. 10.1007/s00251-005-0048-3 16215732

[B10] Kitaoka H. Tsutsui H. Kubo T. Ide T. Chikamori T. Fukuda K. (2021). JCS/JHFS 2018 guideline on the diagnosis and treatment of cardiomyopathies. Circ. J. 85 (9), 1590–1689. 10.1253/circj.CJ-20-0910 34305070

[B11] Marian A. J. Braunwald E. (2017). Hypertrophic cardiomyopathy: genetics, pathogenesis, clinical manifestations, diagnosis, and therapy. Circ. Res. 121 (7), 749–770. 10.1161/CIRCRESAHA.117.311059 28912181 PMC5654557

[B12] Maron B. J. (2018). Clinical course and management of hypertrophic cardiomyopathy. N. Engl. J. Med. 379 (7), 655–668. 10.1056/NEJMra1710575 30110588

[B13] Mclnnes I. B. Schett G. (2011). The pathogenesis of rheumatoid arthritis. N. Engl. J. Med. 365 (23), 2205–2219. 10.1056/nejmra1004965 22150039

[B14] Nakamura H. Tateishi S. Kawakami A. Ida H. Fukuda T. Sasaki M. (2008). A case of mixed connective tissue disease complicated with hypertrophic obstructive cardiomyopathy. Rheumatol. Int. 28 (12), 1273–1275. 10.1007/s00296-008-0608-6 18493766

[B15] Ogawa K. (2016a). Basic knowledge 1 of HLA. Major Histocompat. Complex 23 (2), 115–122. 10.12667/mhc.23.115

[B16] Ogawa K. (2016b). Basic knowledge of HLA part 2. Major Histocompat. Complex 23 (3), 185–192. 10.12667/mhc.23.185

[B17] Ohsuzu F. Katsushika S. Akanuma M. Nakamura H. Harada H. Satoh M. (1997). Hypertrophic obstructive cardiomyopathy due to a novel T-to-A transition at codon 624 in the beta-myosin heavy chain (beta-MHC) gene possibly related to the sudden death. Int. J. Cardiol. 62 (3), 203–209. 10.1016/s0167-5273(97)00256-8 9476679

[B18] Roudier J. (2000). Association of MHC and rheumatoid arthritis. Association of RA with HLA-DR4: the role of repertoire selection. Arthritis Res. 2 (3), 217–220. 10.1186/ar91 11094433 PMC130006

[B19] Teekakirikul P. Eminaga S. Toka O. Alcalai R. Wang L. Wakimoto H. (2010). Cardiac fibrosis in mice with hypertrophic cardiomyopathy is mediated by non-myocyte proliferation and requires Tgf-β. J. Clin. Invest. 120 (10), 3520–3529. 10.1172/JCI42028 20811150 PMC2947222

[B20] Tokunaga K. (2014). Applications of HLA gene polymorphisms. Major Histocompat. Complex 21 (2), 87–95. 10.12667/mhc.21.87

[B21] Yamamoto M. Sato K. Murakoshi N. Yamada Y. Nakagawa D. Nakatsukasa T. (2023). Additional diagnostic value of electron microscopic examination in endomyocardial biopsy in patients with suspected non-ischemic cardiomyopathy. J. Cardiol. 81 (2), 236–243. 10.1016/j.jjcc.2022.09.012 36182004

[B22] Yamanaka H. Sugiyama N. Inoue E. Taniguchi A. Momohara S. (2014). Estimates of the prevalence of and current treatment practices for rheumatoid arthritis in Japan using reimbursement data from health insurance societies and the IORRA cohort (I). Mod. Rheumatol. 24 (1), 33–40. 10.3109/14397595.2013.854059 24261756

